# Disease evolution in mixed connective tissue disease: results from a long-term nationwide prospective cohort study

**DOI:** 10.1186/s13075-017-1494-7

**Published:** 2017-12-21

**Authors:** Silje Reiseter, Ragnar Gunnarsson, Jukka Corander, Joanna Haydon, May Brit Lund, Trond Mogens Aaløkken, Eli Taraldsrud, Siri Opsahl Hetlevik, Øyvind Molberg

**Affiliations:** 1 Institute of Clinical Medicine, University of Oslo, Postbox 1171, Blindern, 0318 Oslo Norway; 2 Department of Rheumatology, Oslo University Hospital, Oslo, Norway; 3 Institute of Basic Medical Sciences, University of Oslo, Oslo, Norway; 4 Department of Rheumatology, Vestre Viken, Drammen, Norway; 5 Department of Respiratory Medicine, Oslo University Hospital, Oslo, Norway; 6 Department of Immunology, Institute for Cancer Research, Oslo University Hospital, Oslo, Norway; 7 Departments of Radiology and Nuclear Medicine, Oslo University Hospital, Oslo, Norway

**Keywords:** Mixed connective tissue disease, Antibodies, Systemic lupus erythematosus, Systemic sclerosis, Anti-ribonucleoprotein, Remission

## Abstract

**Background:**

The phenotypic stability of mixed connective tissue disease (MCTD) is not clear, and knowledge about disease activity and remission is scarce. We aimed to establish the occurrence of evolution from MCTD to another defined rheumatic condition, and the prevalence and durability of remission after long-term observation.

**Methods:**

In this large population-based prospective observational MCTD cohort study (N = 118), disease conversion was defined by the development of new auto-antibodies and clinical features compliant with another well-defined rheumatic condition. Remission was defined by a combination of systemic lupus erythematosus disease activity index 2000 (SLEDAI-2 K) of 0 and European League Against Rheumatism scleroderma trials and research (EUSTAR) activity index <2.5. Predictors of phenotypic stability and disease remission were assessed by logistic regression.

**Results:**

Among 118 patients, 14 (12%) developed another well-defined rheumatic condition other than MCTD after mean disease duration of 17 (SD 9) years. Puffy hands predicted a stable MCTD phenotype in univariable regression analysis (OR 7, CI 2–27, *P* = .010). Disease activity defined by SLEDAI-2 K, decreased gradually across the observation period and > 90% of patients had EUSTAR activity index <2.5. There were 13% patients in remission throughout the whole mean observation period of 7 (SD 2) years. The strongest predictor of remission was percentage of predicted higher forced vital capacity.

**Conclusions:**

Our results strengthen the view of MCTD as a relatively stable disease entity. Long-term remission in MCTD is not frequent; however, the low SLEDAI-2 K and EUSTAR scores during the observation period suggests that the disease runs a milder course than systemic lupus erythematosus and systemic sclerosis.

**Electronic supplementary material:**

The online version of this article (doi:10.1186/s13075-017-1494-7) contains supplementary material, which is available to authorized users.

## Background

Mixed connective tissue disease (MCTD) is a chronic, immune-mediated disorder defined by the combined presence of high titer serum anti-ribonucleoprotein (RNP) antibodies and overlapping features of two or more of the systemic rheumatic disorders: systemic sclerosis (SSc), systemic lupus erythematosus (SLE), polymyositis and rheumatoid arthritis (RA) [1]. MCTD was first presented as a separate disease entity in 1972 by Sharp and colleagues [2]. Since then there has been an ongoing debate about the validity, reliability and appropriateness of the diagnosis; and it has been discussed whether MCTD represents an undifferentiated, transient or stable phenotype. Attempts to resolve these issues have not given consistent results [3–7]. Four MCTD classification criteria have been suggested (Sharp, Kasukawa, Alarcon-Segovia and Kahn) and variably used, possibly resulting in disparate study cohorts. The previous studies assessing the stability of the MCTD phenotype have defined their original patient cohorts by varying criteria [8] and the definitions of cases evolving from MCTD to other systemic rheumatic diseases have differed and not always been clear. The studies by Van den Hoogen [9], Gendi [5] and Ungprasert [7] regarded patients as having evolved from MCTD when they met one or more of the classification criteria for SLE, SSc and RA, thus reporting on patients evolving from MCTD to overlap syndromes. This can be viewed as contradictive when MCTD is defined as a specific overlap syndrome. Nimelstein [10] and Cappelli [6] excluded asymptomatic patients from having MCTD. By applying this strategy, these studies discarded the possibility of attaining remission in MCTD. This is in apparent contrast to Sharp’s original paper [2] where it was stated that MCTD responded well to steroids and had a good prognosis, indicating that many patients attained remission following treatment [2]. However, since there are no specific measures for disease activity in MCTD, remission is not easily defined. Consequently, there is a distinct lack of data on the durability and stability of remission in this disorder.

Consensus on the stability of MCTD would affect future research and the management of patients. Several previous studies have assessed morbidity and mortality risk factors and prognosis in cohorts where, at a defined time point patients fulfilled one or more of the criteria sets for MCTD [11–19]. Ultimately, when clinicians assess an individual patient, the key question is: does the patient belong to a specific risk group?

In this study, we aimed to examine the issue of phenotypic stability, and the prevalence and durability of disease remission in our unselected large population-based MCTD cohort. We assessed phenotypic stability by longitudinal analyses of antibodies and clinical features. Disease remission was defined by absence of disease activity, evaluated by the validated disease activity indexes developed for SSc and SLE [20, 21]. Additionally, we investigated if clinical parameters could predict phenotypic stability and/or disease remission.

## Methods

### Study cohort

We studied 118 patients from the previously described Norwegian nationwide MCTD cohort [22]. The original cohort included 147 patients recruited from all the Departments of Rheumatology in Norway during the years of 2005–2008 [22]. The cohort inclusion criteria were: age above 18 years; fulfillment of at least one of the three sets of criteria for MCTD, the modified Sharp’s criteria [2], the criteria of Alarcón-Segovia [23] and/or Kasukawa [24] and exclusion of another connective tissue disease.

### Clinical data

Patients were assessed at two time points during their disease course: at time point 1 (T1) when the nationwide cohort was established and at time point 2 (T2) for the current study. All patients were examined according to the study protocol at T1. At T2, 87% of the patients were re-examined according to the study protocol. Data were collected by manual review of electronic records (EPR) in the remaining patients. The study protocol included detailed investigations of clinical features, pulmonary function tests (PFT), chest computed tomography (CT), radiographs of the hands, physician’s global assessment (PhGA), routine blood tests and antibody screening. Additionally, these parameters were assessed throughout the observation period by EPR review. Use of the following medications were registered: proton pump inhibitors, nonsteroidal anti-inflammatory drugs (NSAIDs), corticosteroids, azathioprine, methotrexate, cyclophosphamide, mycophenolate, hydroxychloroquine (HCQ), iloprost (a synthetic analog of prostacyclin PGI2), endothelin receptor antagonists, phosphodiesterase 5 inhibitors (PDE5), calcium channel blockers, tumor necrosis factor (TNF) alpha inhibitors and rituximab. Myositis was defined by the parameters applied in the 2 K version of SLE disease activity index (SLEDAI-2 K) [20]: proximal muscle weakness with creatine kinase (CK) elevation, electromyography (EMG) changes or positive muscle biopsy. Radiographs of the hands were assessed for the presence of bone erosions. Interstitial lung disease (ILD) was defined by the presence of pulmonary fibrosis on high-resolution CT as previously described [16]. PFTs were performed according to the American Thoracic Society/European Respiratory Society guidelines [25], using an automated Vmax V6200 system (Sensor Medics, VIASYS Respiratory Care Inc, Yorba Linda, CA, USA). Recorded variables were the percentage of predicted value of forced vital capacity (FVC% pred) and percentage of predicted value of gas diffusing capacity of the lung for carbon monoxide (DLCO% pred). Decreased motility of the esophagus was defined by dynamic x-ray and/or by chest CT (when columns of air and distension that more than doubled esophageal wall thickness were identified). The PhGA was evaluated by a rheumatologist on a scale from 0 to 100 (mm).

### Biochemical parameters, auto-antibodies and HLA typing

Routine blood tests, including hematology, liver and kidney function, erythrocyte sedimentation rate (ESR) and C-reactive protein (CRP) were analyzed at T1 and T2. Additionally, these parameters were assessed throughout the observation period by EPR review. Blood samples drawn at T1 were analyzed for anti-ribonucleoprotein (anti-RNP) using the fully automated fluorescence enzyme immunoassay Phadia 250 (Thermo Fisher Scientific, Phadia GmbH, Freiburg, Germany). We also performed a line immunoassay (ANA Profile 5 Euroline Blot test kit; Euroimmun, Lubeck, Germany) with the three recombined proteins present in the U1-RNP protein complex [26] at T1. HLA typing was performed at T1 as previously described [27]. Blood samples drawn at T2 were analyzed for anti-RNP, anti-histidyl-tRNA synthetase (anti-Jo1), anti-Sjögrens Syndrome antigen A (anti-SSA) and B (anti-SSB), anti-centromere protein-B (ACA), anti-topoisomerase I (anti-scl70) and anti-Smith (anti-Sm) using the same immunoassay (Phadia 250). Anti-RNP values were reported from < 1 to > 240 U/mL. The value of 240 was assigned to all anti-RNP measurements of > 240 U/mL for statistical purposes. Anti-double-stranded DNA (anti-dsDNA) was confirmed by indirect immunofluorescence using *Crithidia luciliae* CLIFT immunofluorescence test (CLIFT) and anti-citrullinated protein antibodies (ACPA) were measured by enzyme-linked immunosorbent assay (ELISA) at T2. Values ten times above the defined cutoff values defined by the laboratory were recorded as strongly positive while values less than three times the cutoff values were recorded as weakly positive. Serum concentrations of C3 and C4 were quantified by nephelometry (Behring, Liederbach, Germany) at T2. Low complement was defined as a C3 and/or C4 count below the lower normal limits: 0.70 g/L for C3 and 0.10 g/L for C4. Thrombocytopenia was defined as < 100 × 10^9^ platelets/L and leukopenia was defined as < 3 × 10^9^ white blood cells (WBC)/L.

### Definition of disease conversion

Patients were defined as having evolution from MCTD when there had been a definite change in the antibody profile together with the occurrence of clinical features compliant with another well-defined rheumatic condition. In cases where more than one specific auto-antibody was identified, the dominant antibody specificity was weighed together with the clinical features.

### Definition of disease remission

There is no validated MCTD disease activity measure or index. The manifestations of MCTD overlap the clinical features of SSc, SLE, idiopathic inflammatory myopathy (IIM) and RA. The SLEDAI-2 K is a validated activity measure for patients with SLE [20]. The preliminary European Scleroderma Trials and Research group (EUSTAR) disease activity index was recently derived and validated in a large SSc cohort [21]. We regarded MCTD activity to be measured appropriately by combining the SLEDAI-2 K and EUSTAR activity index. We considered the myositis and arthritis activity in MCTD patients to be sufficiently measured by the SLEDAI-2 K. In agreement with the recent Definitions of Remission in SLE (DORIS) working group recommendations we defined remission as SLEDAI-2 K = 0 and made the distinction between patients on and off therapy [28]. Remission off therapy required the patient to be on no immune-modulating treatment other than maintenance HCQ. We also allowed for proton pump inhibitors, calcium channel blockers and intermittent use of NSAIDs. Remission on therapy allowed patients to be on low-dose oral corticosteroids (≤5 mg daily) and stable maintenance doses of azathioprine, methotrexate and mycophenolate. The SLEDAI-2 K was measured at two time points (T1, T2) and cumulatively between the two time points. Since the EUSTAR activity index is a measurement of change it was measured at T1 and at T2. Patients with MCTD were defined as being in remission when the SLEDAI-2 K = 0 and the EUSTAR activity index was < 2.5 [21]. As most clinical features in MCTD have a relapsing-remitting pattern, we assessed remission throughout longer time periods in addition to T1 and T2. The term “durable remission” was used to describe patients who were in remission at T1, throughout the observation period and at T2. The term “extended remission” was used to describe patients who had active disease at T1 but achieved remission during the observation period, and were in remission at T2.

### Statistical methods

Groups were compared appropriately using the chi-square test, Fisher’s exact test or one-way analysis of variance (ANOVA) with Tukey’s test or the Kruskal–Wallis test and Mann–Whitney U test for post hoc comparison depending on the distribution. Univariable and multivariable logistic regression analyses were performed to identify predictors of phenotype stability, remission at T2, extended remission and durable remission. Clinical characteristics, age, gender, medications used and remission status at T1 were assessed as possible predictors. Using a manual backward elimination procedure, variables at a significance level of *P* < 0.250 in the univariable analyses were considered candidates for the multivariable predictive model for remission. The prediction was quantified by the odds ratio (OR) with its 95% CI. The Hosmer-Lemeshow goodness-of-fit-test, the Pearson goodness-of-fit test, area under the receiver operating characteristic (ROC) curve, link test, Box–Tidwell test of linearity of the logit and standardized Pearson residuals were all used as measures of fit in the multivariable logistic regression models.

## Results

### Characteristics of the Norwegian MCTD cohort

**Fig. 1:**
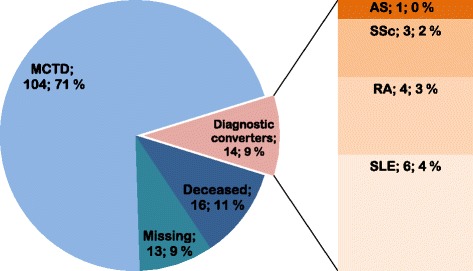
Overview of long-term disease evaluation in the population-based Norwegian mixed connective tissue disease (MCTD) cohort (*N* = 147). ASA, anti-Jo1 positive anti-synthetase syndrome; SSc, systemic sclerosis; RA, rheumatoid arthritis; SLE, systemic lupus erythematosus

Altogether, 16 of the 147 patients from the original nationwide cohort had died before the re-examination at time point T2, while 13 were lost to follow up (Fig. 1). The 13 patients lost to follow up were similar to the rest of the cohort in age, gender distribution and clinical characteristics, while the 16 patients who died before re-examination had older mean age and higher prevalence of pericarditis and ILD (Table 1). Information about the cause of death was available in nine patients. Three patients died of lung cancer, one patient died of pancreas cancer, one patient died from hepato-renal syndrome secondary to alcoholism and two patients died from coronary artery disease. Further, two of the deceased patients had extensive ILD and precapillary pulmonary hypertension (PH). In the remaining seven patients data on the cause of death were unavailable.

**Table 1:** Demographics of the longitudinally observed patients, deceased patients and patients lost to follow up (*N* = 147)

Demographics	Longitudinally observed patients (*N* = 118)	Lost to follow up (*N* = 13)	Deceased before re-examination (*N* = 16)
Age at T1, years, mean (SD)	44 (14)	48 (16)	61 (13)^a^
Disease duration at T1 years, mean (SD)	10 (8)	11 (9)	11 (7)
Male gender, *N* (%)	28 (24)	1 (8)	4 (25)
Anti-RNP, U/mL, median (IQR)	92 (19–240)	91 (33–235)	90 (4–196)
Puffy hands^b^, *N* (%)	108 (92)	12 (92)	14 (88)
Arthritis^b^, *N* (%)	92 (78)	12 (92)	12(75)
Myositis^b^, *N* (%)	36 (30)	3 (23)	6 (38)
Pericarditis^b^, *N* (%)	11 (9)	2 (15)	6 (38)^c^
Sclerodactyly, *N* (%)	33 (28)	6 (46)	5 (31)
Plevritis^b^, *N* (%)	14 (12)	3 (23)	4 (25)
Percentage DLCO pred, mean (SD)	75 (19)	75 (14)	66 (16)
Percentage FVC pred, mean (SD)	93 (18)	88 (19)	88 (24)
Interstitial lung disease, *N* (%)	39 (34)	4 (50)	9 (69)^d^

*T1* time point 1, *Anti-RNP* anti-ribonucleoprotein, *DCLO pred* predicted value of gas diffusing capacity of the lung for carbon monoxide, *FVC pred* predicted value of forced vital capacity

^a^Significant mean age difference between the deceased patients compared to the longitudinally observed patients of 17 years (95% CI 8–26, *P* < .001) and compared to the patients that were lost to follow up: 13 years (95% CI 1–26, *P* = .033)

^b^Ever present at T1

^c^Significantly higher prevalence of pericarditis (*P* = .001) in the deceased patients compared to the longitudinally observed patients

^d^Significantly higher prevalence of interstitial lung disease (*P* = .038) in the deceased patients compared to the longitudinally observed patients

### The prevalence and prediction of phenotype stability

Clinical examinations and auto-antibody profiles analyzed in the 118 longitudinally observed patients demonstrated that 14 patients (9%) had evolved to another specific rheumatic condition during the observation period (Fig. 1; Additional file 1). The mean (SD) observation time between T1 and T2 was 7 (2) years. At T2, the mean disease duration was 17 (9) years. More than 90% of the 118 patients had disease duration > 8 years and were observed for > 5 years. At the time point of re-examination (T2) we found that six patients had shifted to SLE, four to RA, three to SSc and one patient had shifted to anti-Jo1 positive anti-synthetase syndrome (ASA) (Fig. 1; Additional file 1). Clinical and serological parameters of the 104 patients with the stable MCTD phenotype and the 14 diagnostic converters are shown in Table 2 and Additional file 2. At T2, the mean disease duration was 17 (9) years in the 104 patients with a stable MCTD phenotype and 16 (8) years in the 14 diagnostic converters. The mean observation period was 7 (3) in patients with a stable phenotype and 7 (2) years in the diagnostic converters. The cumulative presence of puffy hands at T1 was the only predictor of MCTD phenotype stability at T2 (OR 6.5, CI 1.6–27.1, P = .010) in univariable logistic analyses.

**Table 2:** Clinical parameters of patients with stable MCTD phenotype and diagnostic converters at time point 1

Demographics	Stable MCTD phenotype N = 104	Diagnostic converters N = 14
Age, years, mean (SD)	43 (14)	45 (13)
Disease duration years, mean (SD)	10 (9)	9 (7)
Male gender, *N* (%)	27 (26)	1 (7)
Alarcon-Segovia’s criteria	95 (91)	13 (93)
Kasukawa’s criteria	89 (86)	12 (86)
Sharp’s criteria	101 (97)	13 (93)
Interstitial lung disease, *N* (%)	36 (36)	3 (21)
Cumulative frequency of clinical features		
Puffy hands, *N* (%)	98 (94)	10 (71)^a^
Arthritis, *N* (%)	79 (76)	13 (93)
Myositis, *N* (%)	34 (33)	2 (14)
Raynaud’s phenomenon, *N* (%)	103 (99)	14 (100)
Sclerodactily, *N* (%)	30 (29)	3 (21)
Leukocytopenia, *N* (%)	30 (29)	7 (50)
Thrombocytopenia, *N* (%)	17 (16)	0
Facial erythema, *N* (%)	44 (42)	6 (43)
Pericarditis, *N* (%)	10 (10)	1 (7)
Pleuritis, *N* (%)	13 (13)	1 (7)
Pulmonary function tests		
Percentage DLCO pred, mean (SD)	74 (17)	80 (14)
Percentage FVC pred, mean (SD)	92 (18)	99 (14)
Genetics and anti-RNP antibodies at T1		
Anti-RNP, U/mL, median (IQR)	27 (5–66)	104 (25–240)
HLA DRB1*04:01, *N* (%)	49 (50)	4 (31)

*MCTD* mixed connective tissue disease, *DCLO pred* predicted value of gas diffusing capacity of the lung for carbon monoxide, *FVC pred* predicted value of forced vital capacity, *Anti-RNP* anti-ribonucleoprotein

^a^Significantly greater proportion of patients with puffy hands (*P* = .004) in the stable MCTD phenotype group compared to diagnostic converters

### The prevalence and prediction of remission

**Fig. 2:**
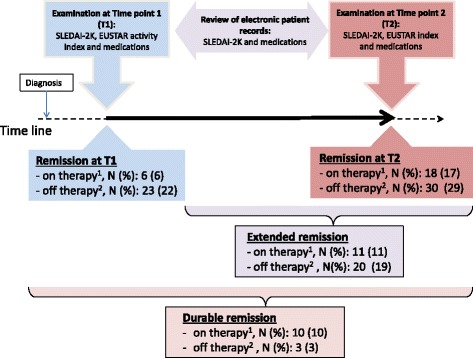
Remission in patients with mixed connective tissue disease (MCTD) on and off therapy (N = 104). ^1^Remission on therapy includes patients using corticosteroids at ≤ 5 mg/day, azathioprine, mycophenolate and methotrexate. ^2^Remission off therapy includes patients using hydroxychloroquine, calcium channel blockers and nonsteroidal anti-inflammatory drugs. T1, timepoint 1; T2, timepoint 2; SLEDAI-2 K, systemic lupus erythematosus disease activity index 2000; EUSTAR, European League Against Rheumatism scleroderma trials and research

Disease activity measured by the median SLEDAI-2 K score decreased with time in the patients with a stable phenotype (N = 104). Correspondingly, the number of patients who had a SLEDAI-2 K score = 0 increased from T1 to T2 (Additional file 3). The majority of patients with active disease had arthritis and rash, followed by alopecia (Additional file 3). These manifestations were all found to decrease over time, differing from leukopenia that fluctuated and thrombocytopenia that was persistent in the affected patients. There were two new cases of onset of trigeminal neuropathy between T1 and T2 and one of persistent myositis (Additional file 3). When disease activity was measured by the EUSTAR activity index recently developed for SSc, we found that less than 10% of the patients with MCTD had active disease (Additional file 4). The prevalence of digital ulcers decreased from T1 to T2, while the prevalence of patients with DLCO % pred <70 increased slightly, and was evident in 42% of patients at T2 (Additional file 4). The number of patients in remission (defined as a SLEDAI-2 K score = 0 and EUSTAR activity index <2.5) during the observation period is shown in Fig. 2. In total 13 patients (13%) met the definition of “durable remission”, while 31 patients (30%) met the definition of extended remission and 48 patients (46%) were in remission at T2 (Fig. 2 and Additional file 5 show patients in remission on and off therapy and patients excluded from the remission group due to medications not compatible with remission). We observed that two patients with ILD progression and one with progressive pulmonary arterial hypertension (PAH) were classified by the EUSTAR activity index as having inactive disease, but due to their medications (cyclophosphamide and endothelin receptor antagonist) the three patients were not defined as being in remission. We found that the median PhGA was lower in the durable remission group than in the patient groups with shorter duration of remission (Additional file 5).

We assessed possible predictors of the three remission groups (remission at T2, extended remission and durable remission). In both univariable (Additional file 6) and multivariable analyses higher FVC % pred at T1 enhanced the odds of achieving and remaining in remission (Table 3). Patients with thrombocytopenia ever present at T1 and patients using NSAIDs at T1 were less likely to be in both extended remission and remission at T2, in univariable analyses (Additional file 6). The strongest predictors of being in remission at the time of re-examination (T2) were high FVC % pred and never having thrombocytopenia. In the multivariable model the strongest predictors of extended remission were higher FVC % pred, elevated CK and never having digital ulcers. The strongest predictors of durable remission were negative anti-RNP test, higher FVC % pred, never having facial erythema and not using NSAIDs (Table 3).

**Table 3:** Predicting models of remission at time point 2, extended remission and durable remission by multivariable logistic regression analyses (*N* = 104 patients)

Clinical features present at T1	Model 1: remission^a^ at T2 (*N* = 48)			Model 2: extended remission^a^ (N = 31)			Model 3: durable remission^a^ (*N* = 13)		
	OR	95% CI	*P* value	OR	95% CI	*P* value	OR	95% CI	*P* value
Increased CK^b^	-	-	-	3.18	1.17–8.67	.024*	-	-	-
Facial erythema^b^	-	-	-	-	-	-	0.11	0.01–0.81	.030*
Digital ulcers^b^	-	-	-	0.25	0.08–0.77	.015*	-	-	-
Trombocytophenia^b,^ ^c^	0.05	0.01–-0.44	.006**	-	-	-	-	-	-
FVC % pred (pr 10%)	1.37	1.04–1.79	.026*	1.56	1.13–2.16	.007**	1.71	1.04–2.80	.033*
NSAID medication	-	-	-	-	-	-	0.05	0.00–0.73	.028*
Negative anti-RNP	-	-	-	-	-	-	15.0	1.97–113.59	.009**

Area under receiver operating curve (ROC) was 73% for Model 1, 76% for Model 2 and 83% for Model 3

*T1* time point 1, *T2* time point 2, *CK* creatine kinase, *FVC % pred* percentage of predicted forced vital capacity, *NSAIDs* nonsteroidal anti-inflammatory drugs, *anti-RNP* anti-ribonucleoprotein, *ROC* receiver operating characteristic

* *P* <.05; ** *P* <.01

^a^Includes patients in remission both on and off therapy; medications allowed were hydroxychloroquine, NSAIDs, corticosteroids at ≤ 5 mg daily, calcium channel blockers, methotrexate, mycophenolate and azathioprine

^b^Ever present at T1

^c^Thrombocytes <100 × 10^9^ platelets/L

## Discussion

Phenotype stability in MCTD has been an ongoing debate for many years, but has rarely been addressed by large, population-based longitudinal studies. In this study we showed that the phenotype appears stable in the majority of patients and even though durable remission was rare, the outcome was favorable in terms of declining disease activity over time. These results provide important new knowledge on long-term outcome and should have an impact on the management of patients with MCTD.

Defining a clinical entity based on auto-antibody specificity was viewed as controversial in 1972, but the continuing discoveries of relevant auto-antibody analyses for diagnostic and risk classification purposes [29, 30] may possibly prove its value. Hence, we reasoned that it was rational to examine disease conversion by investigating a change in auto-antibody status together with clinical features in patients with MCTD. By this approach, we identified 14 converters, 5 of whom shifted to SLE and had high titers of anti-dsDNA and low levels of complement. The second largest group of converters developed ACPA positivity and were defined as RA converters. Hand radiographs revealed that all four patients in this group had bone erosions. This was only seen in nine other patients from the original MCTD cohort. Thus, bone erosions are uncommon in MCTD and should raise a query about the risk of group change. Three patients had shifted to SSc with the development of ACA. One of these patients had a complex phenotype with high titers of anti-SSA and anti-SSB and subsequent mucosa-associated lymphatic tissue (MALT) lymphoma. Importantly, there was no enrichment of patients with multiple auto-antibodies in the cohort. The cumulative manifestation of puffy hands at T1 was associated with MCTD phenotypic stability, possibly indicating that the manifestation should be included if a unified MCTD classification criteria set was to be used.

Nearly half of the patients with MCTD were in remission at the time of re-examination at T2, but only 13% had been in sustained remission throughout the whole observation period. The SLEDAI-2 K scores fell across the observation period, equally to what has been reported in SLE cohorts [31]. We identified lower SLEDAI-2 k scores in our cohort than were found by Carpintero et al. in 2015 [32]; this could be due to differences in cohort populations and disease duration. The SLEDAI-2 K scores also appeared lower than described in SLE cohorts [33]. In addition, the EUSTAR activity index scores were lower in our MCTD cohort than reported in the SSc cohort used for index validation [21]. The ILD prevalence was less than in SSc cohorts [16] and our research group have previously shown that the percentage of total lung volume affected is less than reports from SSc cohorts [34]. Taken together, these results suggest that MCTD involves milder disease activity than SSc and SLE. There are however some concerns: mortality rates have been reported to be high in MCTD [14] and it is known from SLE studies that organ damage increase over time even though disease activity decreases [35]. Organ damage over time in MCTD should be assessed further in future studies.

Negative anti-RNP antibodies were a predictor of durable remission in the multivariable model, but not a predictor of the other remission groups. Thus, we did not find negative anti-RNP antibodies to be as strongly associated with remission as Burdt et al. reported in 1999 [36]. Reasons for this might include the small number of patients with negative anti-RNP antibodies and the strict definition of remission. The strongest predictor of remission at all time points was higher FVC % pred at T1, possibly reflecting a connection between lung disease and MCTD disease activity.

One of the limitations of this study was that 9% of the original nationwide cohort was lost to follow up and 11% had died before re-examination (T2). However it appeared that patients lost to follow up did not differ significantly from the rest of the cohort. Another limitation was incomplete clinical and antibody profiles of the patients that died before re-examination (11%). By reviewing clinical information and cause of death in these patients we lowered the possibility of selection bias. Additionally, diagnostic conversion and durable remission was found to be rare, thus weakening the predictor findings. Major strengths of the study are (1) the long-term observation period, (2) the protocol-based examinations at two time points (in addition to thorough EPR reviews), (3) results from a large MCTD population and (4) minimal selection bias (unselected nationwide MCTD cohort). Additional strengths were strict definitions of disease conversion, and application of validated remission criteria derived from two diseases with features overlapping those of MCTD.

## Conclusion

The results of this study indicate that the majority of patients with MCTD retain their phenotype during long-term observation and MCTD involves milder disease activity than SSc and SLE.

## Additional files

Additional file 1: Clinical and laboratory features in the patients evolving from MCTD to another specific rheumatic condition. (PDF 157 kb)

Additional file 2: Auto-antibodies at time point 2 in 118 patients with MCTD. (PDF 154 kb)

Additional file 3: SLEDAI-2 K in 104 patients with MCTD at time point 1 and 2. (PDF 19 kb)

Additional file 4: EUSTAR activity index in 104 patients with MCTD at time point 1 and 2. (PDF 149 kb)

Additional file 5: Remission and medications in patients with mixed connective tissue disease, N = 104. Patients with SLEDAI-2 K = 0 and EUSTAR activity index ≥2.5 were first selected, then excluded if using medication not compatible with remission and finally sub grouped in remission on and off therapy. (PDF 128 kb)

Additional file 6: Univariable logistic regression analyses for remission at time point 2, “extended remission” and “durable remission”. (PDF 194 kb)

## Abbreviations

ACA: Anti-centromere protein-B
ACPA: Anti-citrullinated protein antibodies
anti-dsDNA: Anti-double stranded DNA
anti-Jo1: Anti-histidyl-tRNA synthetase
anti-scl70: Anti-topoisomerase I
anti-Sm: Anti-Smith
anti-SSA: Anti-Sjögrens Syndrome antigen A
anti-SSB: Anti-Sjögrens Syndrome antigen B
CK: Creatine kinase
CLIFT: *Crithidia luciliae* immunofluorescence test
CRP: C-reactive protein
CT: Computed tomography
DLCO% pred: Percentage of predicted value of gas diffusing capacity of the lung for carbon monoxide
DORIS: Definitions of remission in SLE
ELISA: Enzyme-linked immunosorbent assay
EMG: Electromyography
EPR: Electronic patient records
ESR: Erythrocyte sedimentation rate
EUSTAR: European League Against Rheumatism scleroderma trials and research
FVC% pred: Percentage of predicted value of forced vital capacity
HCQ: Hydroxychloroquine
ILD: Interstitial lung disease
MCTD: Mixed connective tissue disease
NSAIDs: Nonsteroidal anti-inflammatory drugs
OR: Odds ratio
PDE5: Phosphodiesterase 5 inhibitors
PFT: Pulmonary function tests
PH: Ppulmonary hypertension
PhGA: Physician’s global assessment
RA: Rheumatoid arthritis
RNP: Ribonucleoprotein
SLE: Systemic lupus erythematosus
SLEDAI-2 K: Systemic lupus erythematosus disease activity index 2000
SSc: Systemic sclerosis
T1: Time point 1
T2: Time point 2
TNF: Tumor necrosis factor

## References

[1] Tani C, Carli L, Vagnani S, Talarico R, Baldini C, Mosca M, Bombardieri S (2014). The diagnosis and classification of mixed connective tissue disease. J Autoimmun.

[2] Sharp GC, Irvin WS, Tan EM, Gould RG, Holman HR (1972). Mixed connective tissue disease–an apparently distinct rheumatic disease syndrome associated with a specific antibody to an extractable nuclear antigen (ENA). Am J Med.

[3] Moore OA, Proudman SM, Goh N, Corte TJ, Rouse H, Hennessy O, Morrisroe K, Thakkar V, Sahhar J, Roddy J (2015). Quantifying change in pulmonary function as a prognostic marker in systemic sclerosis-related interstitial lung disease. Clin Exp Rheumatol.

[4] van den Hoogen F, Khanna D, Fransen J, Johnson SR, Baron M, Tyndall A, Matucci-Cerinic M, Naden RP, Medsger TA, Carreira PE (2013). Classification criteria for systemic sclerosis: an American College of Rheumatology/European League Against Rheumatism collaborative initiative. Ann Rheum Dis.

[5] Gendi NS, Welsh KI, Van Venrooij WJ, Vancheeswaran R, Gilroy J, Black CM (1995). HLA type as a predictor of mixed connective tissue disease differentiation. Ten-year clinical and immunogenetic follow up of 46 patients. Arthritis Rheum.

[6] Cappelli S, Bellando Randone S, Martinovic D, Tamas MM, Pasalic K, Allanore Y, Mosca M, Talarico R, Opris D, Kiss CG (2012). “To be or not to be”, ten years after: evidence for mixed connective tissue disease as a distinct entity. Semin Arthritis Rheum.

[7] Ungprasert P, Crowson CS, Chowdhary VR, Ernste FC, Moder KG, Matteson EL (2016). Epidemiology of mixed connective tissue disease 1985-2014: a population based study. Arthritis Care Res (Hoboken).

[8] Frandsen PB, Kriegbaum NJ, Ullman S, Hoier-Madsen M, Wiik A, Halberg P (1996). Follow-up of 151 patients with high-titer U1RNP antibodies. Clin Rheumatol.

[9] van den Hoogen FH, Spronk PE, Boerbooms AM, Bootsma H, de Rooij DJ, Kallenberg CG, van de Putte LB (1994). Long-term follow-up of 46 patients with anti-(U1)snRNP antibodies. Br J Rheumatol.

[10] Nimelstein SH, Brody S, McShane D, Holman HR (1980). Mixed connective tissue disease: a subsequent evaluation of the original 25 patients. Medicine (Baltimore).

[11] Fagundes MN, Caleiro MT, Navarro-Rodriguez T, Baldi BG, Kavakama J, Salge JM, Kairalla R, Carvalho CR (2009). Esophageal involvement and interstitial lung disease in mixed connective tissue disease. Respir Med.

[12] Hetlevik SO, Flato B, Rygg M, Nordal EB, Brunborg C, Hetland H, Lilleby V. Long-term outcome in juvenile-onset mixed connective tissue disease: a nationwide Norwegian study. Ann Rheum Dis. 2016;76(1):159–65. http://ard.bmj.com/content/76/1/159.long. 10.1136/annrheumdis-2016-209522 27283334

[13] Ungprasert P, Wannarong T, Panichsillapakit T, Cheungpasitporn W, Thongprayoon C, Ahmed S, Raddatz DA (2014). Cardiac involvement in mixed connective tissue disease: a systematic review. Int J Cardiol.

[14] Hajas A, Szodoray P, Nakken B, Gaal J, Zold E, Laczik R, Demeter N, Nagy G, Szekanecz Z, Zeher M (2013). Clinical course, prognosis, and causes of death in mixed connective tissue disease. J Rheumatol.

[15] Gunnarsson R, El-Hage F, Aalokken TM, Reiseter S, Lund MB, Garen T, Norwegian M, Molberg O (2016). Associations between anti-Ro52 antibodies and lung fibrosis in mixed connective tissue disease. Rheumatology (Oxford).

[16] Reiseter S, Gunnarsson R, Mogens Aalokken T, Brit Lund M, Mynarek G, Corander J, Haydon J, Molberg O. Progression and mortality of interstitial lung disease in mixed connective tissue disease: a long-term observational nationwide cohort study. Rheumatology (Oxford). 2017. https://doi.org/10.1093/rheumatology/kex077. 10.1093/rheumatology/kex077 28379478

[17] Gunnarsson R, Andreassen AK, Molberg O, Lexberg AS, Time K, Dhainaut AS, Bertelsen LT, Palm O, Irgens K, Becker-Merok A (2013). Prevalence of pulmonary hypertension in an unselected, mixed connective tissue disease cohort: results of a nationwide, Norwegian cross-sectional multicentre study and review of current literature. Rheumatology (Oxford).

[18] Szodoray P, Hajas A, Kardos L, Dezso B, Soos G, Zold E, Vegh J, Csipo I, Nakken B, Zeher M (2012). Distinct phenotypes in mixed connective tissue disease: subgroups and survival. Lupus.

[19] Lundberg IE (2005). The prognosis of mixed connective tissue disease. Rheum Dis Clin North Am.

[20] Gladman DD, Ibanez D, Urowitz MB (2002). Systemic lupus erythematosus disease activity index 2000. J Rheumatol.

[21] Valentini G, Iudici M, Walker UA, Jaeger VK, Baron M, Carreira P, Czirjak L, Denton CP, Distler O, Hachulla E (2017). The European Scleroderma Trials and Research group (EUSTAR) task force for the development of revised activity criteria for systemic sclerosis: derivation and validation of a preliminarily revised EUSTAR activity index. Ann Rheum Dis.

[22] Gunnarsson R, Molberg O, Gilboe IM, Gran JT, Group PS (2011). The prevalence and incidence of mixed connective tissue disease: a national multicentre survey of Norwegian patients. Ann Rheum Dis.

[23] Alarcon-Segovia D, Cardiel MH (1989). Comparison between 3 diagnostic criteria for mixed connective tissue disease. Study of 593 patients. J Rheumatol.

[24] Kasukawa RS, Gordon C. Mixed connective tissue disease and anti-nuclear antibodies: proceedings of the International Symposium on Mixed Connective Tissue Disease and Anti-nuclear Antibodies, Tokyo, 29-30 August 1986/editors, Reiji Kasukawa, Gordon C. Sharp. In International Symposium on Mixed Connective Tissue Disease and Anti-nuclear Antibodies 1986: Tokyo, Japan: Amsterdam; New York: Excerpta Medica; New York, NY, USA: Elsevier Science Pub. Co.; 1987

[25] Miller MR, Hankinson J, Brusasco V, Burgos F, Casaburi R, Coates A, Crapo R, Enright P, van der Grinten CP, Gustafsson P (2005). Standardisation of spirometry. Eur Respir J.

[26] Gunnarsson R, Hetlevik SO, Lilleby V, Molberg O (2016). Mixed connective tissue disease. Baillieres Best Pract Res Clin Rheumatol.

[27] Flam ST, Gunnarsson R, Garen T, Lie BA, Molberg O, Norwegian Mctd Study Group (2015). The HLA profiles of mixed connective tissue disease differ distinctly from the profiles of clinically related connective tissue diseases. Rheumatology (Oxford)..

[28] van Vollenhoven R, Voskuyl A, Bertsias G, Aranow C, Aringer M, Arnaud L, Askanase A, Balazova P, Bonfa E, Bootsma H (2017). A framework for remission in SLE: consensus findings from a large international task force on definitions of remission in SLE (DORIS). Ann Rheum Dis.

[29] Ghirardello A, Borella E, Beggio M, Franceschini F, Fredi M, Doria A (2014). Myositis autoantibodies and clinical phenotypes. Auto Immun Highlights.

[30] Domsic RT (2014). Scleroderma: the role of serum autoantibodies in defining specific clinical phenotypes and organ system involvement. Curr Opin Rheumatol.

[31] Wilhelm TR, Magder LS, Petri M (2017). Remission in systemic lupus erythematosus: durable remission is rare. Ann Rheum Dis.

[32] Carpintero MF, Martinez L, Fernandez I, Romero AC, Mejia C, Zang YJ, Hoffman RW, Greidinger EL (2015). Diagnosis and risk stratification in patients with anti-RNP autoimmunity. Lupus.

[33] Uribe AG, Vila LM, McGwin G, Sanchez ML, Reveille JD, Alarcon GS (2004). The Systemic Lupus Activity Measure-revised, the Mexican Systemic Lupus Erythematosus Disease Activity Index (SLEDAI), and a modified SLEDAI-2 K are adequate instruments to measure disease activity in systemic lupus erythematosus. J Rheumatol.

[34] Hoffmann-Vold AM, Aalokken TM, Lund MB, Garen T, Midtvedt O, Brunborg C, Gran JT, Molberg O. Predictive value of serial HRCT analyses and concurrent lung function tests in systemic sclerosis. Arthritis Rheumatol. 2015;67(8):2205–12. 10.1002/art.39166 25916462

[35] Urowitz MB, Gladman DD, Ibanez D, Fortin PR, Bae SC, Gordon C, Clarke A, Bernatsky S, Hanly JG, Isenberg D (2012). Evolution of disease burden over five years in a multicenter inception systemic lupus erythematosus cohort. Arthritis Care Res (Hoboken).

[36] Burdt MA, Hoffman RW, Deutscher SL, Wang GS, Johnson JC, Sharp GC (1999). Long-term outcome in mixed connective tissue disease: longitudinal clinical and serologic findings. Arthritis Rheum.

