# Supplementary material for: Disease evolution in mixed connective tissue disease: results from a long-term nationwide prospective cohort study
Source: Arthritis Res Ther. 2017 Dec 21;19:284. doi: 10.1186/s13075-017-1494-7 (PMC5740892; doi:10.1186/s13075-017-1494-7)
Supplement: Supplementary file 1 — Clinical and laboratory features in the patients evolving from MCTD to another specific rheumatic condition. (PDF 157 kb) [file 13075_2017_1494_MOESM1_ESM.pdf]

Additional file 1: Clinical and laboratory features in the patients evolving into another specific rheumatic condition

Case	Time point 1	Time point 2
1	RP, puffy hands, arthritis, leukopenia	Arthritis[1], elevated CK, DLCO % pred < 70[2], strongly positive anti-dsDNA and anti-SSA, low C3 and C4, negative anti-RNP
2	RP, puffy hands, pericarditis, pleuritis, leukopenia, oesophageal dysmotility, facial erythema	RP, pericarditis, puffy hands, sclerodactyly, oesophageal dysmotility, arthritis, positive anti-dsDNA and anti-RNP, low C3 and C4
3	RP, puffy hands, arthritis, lymphadenitis, facial erythema, leukopenia, oesophageal dysmotility	RP, puffy hands, arthritis, lymphadenitis, facial erythema, leukopenia, thrombocytopenia, nephritis, positive anti-dsDNA, anti-SSA and anti-RNP, low C3 and C4
4	RP, puffy hands, arthritis - facial erythema, digital ulcers, muscle weakness, lymphadenopathy, ILD	RP, puffy hands, arthritis, lymphadenopathy, alopecia, ILD, strongly positive anti-dsDNA, anti-Sm, anti-SSA and anti-RNP, low C3 and C4
5	RP, facial erythema, arthritis, leukopenia, oesophageal dysmotility	RP, arthritis, oesophageal dysmotility, anaemia, pericarditis, pleuritic, positive anti-RNP, strongly positive anti-dsDNA and anti-SSA, low C3 and C4
6	RP, puffy hands, leukopenia, anaemia, arthritis, facial erythema, lymphadenopathy	RP, puffy hands - DLCO % pred < 70[2], leukopenia, positive anti-RNP, strongly positive anti-dsDNA, low C3 and C4
7	RP, puffy hands, leukopenia, anaemia, arthritis	RP, arthritis, bone erosion, negative anti-RNP, strongly positive ACPA
8	RP, arthritis, leukopenia, puffy hands, anaemia	Bone erosion, arthritis, strongly positive ACPA, negative ANA
9	RP, facial erythema, alopecia, elevated CK, arthritis	RP, elevated CK, bone erosion, arthritis, strongly positive ACPA, negative ANA
10	RP, muscle weakness, facial erythema, alopecia, arthritis	RP, arthritis, bone erosions, negative RNP, strongly positive ACPA and RF IgM
11	RP, arthritis, alopecia, sclerodactyly, myositis	RP, MALT-lymphoma, arthritis, lymphadenopathy, sclerodactyly, IL, negative anti-RNP, strongly positive ACA, anti-SSB and anti-SSA, positive anti-Jo1
12	RP, DLCO % pred < 70[2], puffy hands, sclerodactyly, arthritis, digital ulcers	RP, puffy hands, sclerodactyly, digital ulcers, DLCO % pred < 70, strongly positive ACA, positive anti-RNP
13	RP, oesophageal dysmotility, puffy hands, sclerodactyly, alopecia, arthritis, facial erythema, digital ulcers, muscle weakness, lymphadenopathy, ILD	RP, puffy hands, sclerodactyly, muscle weakness, lymphadenopathy, ILD, strongly positive ACA, positive anti-Scl70, negative anti-RNP
14	RP, puffy hands, arthritis, oesophageal dysmotility, myositis, ILD	Myositis, puffy hands, oesophageal dysmotility, arthritis, ILD, bone erosion, strongly positive anti-Jo1, positive anti-SSA and anti-RNP

[1] no bone erosions on hand X-ray, [2] no ILD on CT, RP: Raynaud's phenomenon, ILD: Interstitial Lung Disease, SSc: Systemic sclerosis, ASS: Antisynthetase Syndrome, SLE: Systemic Lupus Erythematosus, anti-Jo1: anti-histidyl-tRNA synthetase, anti-SSA: anti-Sjögrens Syndrome antigen A, anti-SSB: anti-Sjögrens Syndrome antigen B, ACA: anti-centromere protein-B, anti-scl70: anti-topoisomerase I, anti-Sm: anti-Smith, anti-dsDNA: Anti-double stranded DNA and ACPA; Anti-citrullinated protein antibodies.
