# Supplementary material for: Disease evolution in mixed connective tissue disease: results from a long-term nationwide prospective cohort study
Source: Arthritis Res Ther. 2017 Dec 21;19:284. doi: 10.1186/s13075-017-1494-7 (PMC5740892; doi:10.1186/s13075-017-1494-7)
Supplement: Supplementary file 2 — Auto-antibodies at time point 2 in 118 patients with MCTD. (PDF 154 kb) [file 13075_2017_1494_MOESM2_ESM.pdf]

Additional file 2: Auto-antibodies at Time point 2 in 118 MCTD patients

**Antibody status at time point 2***	**Stable MCTD phenotype**<br><br>**N = 104**	**Diagnostic converters**<br><br>**N = 14**
Anti-RNP antibodies	78 (75)	7 (50)
Anti-RNP, U/mL, Mdn (IQR)	61 (9 – 240)	15 (3 – 37)
Anti-dsDNA antibodies**	3 (3)	6 (43)
Anti-Sjögrens Syndrome antigen A (anti-SSA)	18 (17)	6 (43)
Anti-Sjögrens Syndrome antigen B (anti-SSB)	4 (4)	1 (7)
Anti-topoisomerase I (anti-scl70)	0	1 (7)
Anti-centromere protein-B (ACA)	0	3 (21)
Anti-histidyl-tRNA synthetase (anti-Jo1)	0	2 (14)
Anti-Smith (anti-Sm)**	7 (7)	1 (7)
Low complements	6 (6)	6 (43)
Anti-citrullinated protein antibodies (ACPA)**	5 (5)	4 (29)

*presented as N (%) unless otherwise specified. **The patients categorized as stable MCTD patients had only weakly positive anti-dsDNA antibodies, anti-Smith antibodies or anti-citrullinated protein antibodies, combined with strongly positive anti-RNP antibodies in addition to MCTD features. Weakly positive anti-dsDNA antibodies, anti-Smith antibodies or low complements did not occur together in the patients categorized as stable MCTD patients.
