# Supplementary material for: Disease evolution in mixed connective tissue disease: results from a long-term nationwide prospective cohort study
Source: Arthritis Res Ther. 2017 Dec 21;19:284. doi: 10.1186/s13075-017-1494-7 (PMC5740892; doi:10.1186/s13075-017-1494-7)
Supplement: Supplementary file 3 — SLEDAI-2 K in 104 patients with MCTD at time point 1 and 2. (PDF 19 kb) [file 13075_2017_1494_MOESM3_ESM.pdf]

Additional file 3: SLEDAI-2K in 104 MCTD patients at Time point 1 and 2

Descriptor	T1<br>(last 10 days)	Between T1 and T2	T2<br>(last 10 days)
Recent onset seizure, N (%)	0	0	0
Psychosis, N (%)	0	0	0
Organic Brain Syndrome, N (%)	0	0	0
Visual Disturbance, N (%)	0	0	0
New onset Cranial Nerve Disorder , N (%)	0	2 (2)	0
Lupus Headache, N (%)	0	0	0
New cerebrovascular accident, N (%)	0	0	0
Vasculitis, N (%)	0	0	0
Arthritis, N (%)	26 (25)	20 (19)	11 (11)
Myositis, N (%)	8 (8)	1 (1)	1 (1)
Urinary casts, N (%)	0	2 (2)	0
Hematuria, N (%)	0	4 (4)	0
Proteinuria, N (%)	1	4 (4)	1 (1)
Pyuria, N (%)	0	3 (3)	0
Rash, N (%)	29 (28)	20 (19)	16 (15)
Alopecia, N (%)	13 (13)	3 (3)	4 (4)
Mucosal ulcers, N (%)	0	4 (4)	0
Pleurisity, N (%)	0	6 (6)	0
Pericarditis, N (%)	1 (1)	4 (4)	0
Low complements, N (%)	7 (7)	0	6 (6)
Positive dsDNA, N (%)	3 (3)	0	3 (3)
Fever, N (%)	0	0	0
Thrombocytopenia, N (%)	4 (4)	4 (4)	5 (5)
Leukopenia, N (%)	7 (7)	12 (12)	3 (3)
SLEDAI-2K score, median (Q1-Q3)	2 (0-4)	1 (0-4)	0 (0-2)
SLEDAI-2K = 0, N (%)	36 (35)	50 (48)	63 (61)

T1: Time point 1, T2: Time point 2
