# Supplementary material for: Disease evolution in mixed connective tissue disease: results from a long-term nationwide prospective cohort study
Source: Arthritis Res Ther. 2017 Dec 21;19:284. doi: 10.1186/s13075-017-1494-7 (PMC5740892; doi:10.1186/s13075-017-1494-7)
Supplement: Supplementary file 4 — EUSTAR activity index in 104 patients with MCTD at time point 1 and 2. (PDF 149 kb) [file 13075_2017_1494_MOESM4_ESM.pdf]

Additional file 4: EUSTAR activity index in 104 MCTD patients at Time point 1 and 2

Descriptor	T1	T2
Δ-skin, N (%)	0	0
Digital Ulcers, N (%)	15 (14)	10 (10)
mRSS > 18, N (%)	0	0
TFR, N (%)	0	0
CRP > 1 mg/dL, N (%)	11 (11)	8 (8)
DLCO % pred < 70, N (%)	35 (34)	44 (42)
Eustar activity index, Median (IQR)	0 (0 – 1)	0 (0 – 1)
Eustar activity index < 2.5	96 (92)	97 (93)

T1: Time point 1, T2: Time point 2, TFR: tendon friction rub, mRSS: modified Rodnan Skin Score, CRP: C-Reactive Protein, DLCO % pred: Diffusing Capacity for Carbon Monoxide percentage of predicted, N: number, IQR: interquartile range
