# Supplementary material for: Disease evolution in mixed connective tissue disease: results from a long-term nationwide prospective cohort study
Source: Arthritis Res Ther. 2017 Dec 21;19:284. doi: 10.1186/s13075-017-1494-7 (PMC5740892; doi:10.1186/s13075-017-1494-7)
Supplement: Supplementary file 5 — Remission and medications in patients with mixed connective tissue disease, N = 104. Patients with SLEDAI-2 K = 0 and EUSTAR activity index ≥2.5 were first selected, then excluded if using medication not compatible with remission and finally sub grouped in remission on and off therapy. (PDF 128 kb) [file 13075_2017_1494_MOESM5_ESM.pdf]

Additional file 5: Remission and medication in Mixed Connective Tissue Disease patients, N = 104. Patients with SLEDAI-2K = 0 and EUSTAR activity index ≥ 2.5 were first selected, then excluded if using medication not compatible with remission and finally sub grouped in remission on and off therapy.

**MCTD patients, N (%)**	**T1**	**T2**	**Extended**	**Durable**
**SLEDAI-2K = 0 and ACR/EUSTAR activity index < 2.5**	**34 (33)**	**59 (57)**	**38 (37)**	**17 (16)**
**On medications not compatible with remission:**	**5 (15)**	**11 (19)**	**7 (18)**	**4 (24)**
Oral corticosteroids > 5 mg daily	5 (15)	9 (15)	3 (8)	2 (12)
Methylprednisolone intravenously	0	0	2 (5)	1 (6)
Iloprost	0	1 (2)	1 (3)	1 (6)
Endothelin receptor antagonist (bosentan)	0	1 (2)	1 (3)	1 (6)
Rituximab	0	1 (2)	1 (3)	0
Cyclophosphamide	0	0	2 (6)	1 (6)
**Total remission: (On and off therapy)**	**29 (28)**	**48 (46)**	**31 (30)**	**13 (13)**
PhGA, median (Q1 – Q3)	NA	13 (8 - 25)	10 (5 - 21)	8 (4 - 12)
**On therapy**	**6 (6)**	**18 (17)**	**11 (11)**	**3 (3)**
Oral steroids ≤ 5 mg daily	6 (100)	12 (67)	8 (73)	2 (67)
AZA	1 (17)	3 (17)	3 (27)	0
MTX	2 (33)	5 (28)	3 (27)	1 (33)
MMF	0	2 (11)	1 (10)	0
**Off therapy**	**23 (22)**	**30 (29)**	**20 (19)**	**10 (10)**
HCQ	6 (26)	9 (30)	6 (30)	1 (10)
Proton pump inhibitors	6 (26)	9 (30)	4 (20)	0
Calcium channel blockers	6 (26)	6 (20)	4 (20)	2 (20)
NSAIDs	4 (17)	6 (20)	4 (20)	2 (20)

PGA – Physicians Global Assessment (0-100 mm), HCQ – Hydroxychloroquine, MTX –methotrexate, MMF – mycophenolate, AZA – azathioprine. On therapy allowed for low-dose oral corticosteroids (≤ 5 mg daily) and stable maintenance doses of azathioprine, methotrexate and mycophenolate. Off therapy allowed for hydroxychloroquine, proton pump inhibitors, calcium channel blockers and intermittent use of NSAID’s.
