# Supplementary material for: Disease evolution in mixed connective tissue disease: results from a long-term nationwide prospective cohort study
Source: Arthritis Res Ther. 2017 Dec 21;19:284. doi: 10.1186/s13075-017-1494-7 (PMC5740892; doi:10.1186/s13075-017-1494-7)
Supplement: Supplementary file 6 — Univariable logistic regression analyses for remission at time point 2, “extended remission” and “durable remission”. (PDF 194 kb) [file 13075_2017_1494_MOESM6_ESM.pdf]

Additional file 6: Univariable logistic regression analyses for remission at Time point 2, "extended remission" and "durable remission"

Clinical features and treatment at T1	Remission at follow-up (T2)[1] (n=48)			Extended remission[1] (n= 31)			Durable remission[1] (n=13)		
	OR	95% CI	P value	OR	95 % CI	P value	OR	95 % CI	P value
Muscle weakness[2]	.51	.23 - 1.13	.095	-	-	>.250	.19	.04 -.93	.040*
Elevated CK levels[2]	2.30	1.02 - 5.17	.044*	2.32	.98-5.48	.055	-	-	>.250
Facial erythema[2]	.50	.23 – 1.11	.088	-	-	>.250	.21	.04 - 1.10	.052
Alopecia[2]	.49	.20 – 1.22	.124	-	-	>.250	.21	.03- 1.68	.141
Digital ulcers[2]	-	-	>.250	.31	.11-.84	.021*	-	-	>.250
Pleuritis[2]	-	-	>.250	.39	.08 – 1.87	.238	-	-	>.250
Pericarditis[2]	-	-	>.250	.24	.03 – 1.96	.181	-	-	>.250
FVC % pred (pr 10%)	1.37	1.06 – 1.78	.017*	1.50	1.12 – 2.03	.007**	1.54	1.03 -2.31	.036
Thrombocytopenia[2]	.053	.01 - .42	.005**	.12	.02 - .94	.043*	-	-	>.250
Remission at T1[1]	2.73	1.04 – 7.16	.042*	2.20	.84 – 5.76	.109	-	-	NA
NSAID	.33	.14 - .81	.016*	.33	.11 - .95	.041*	.16		.086
Calcium channel blockers	-	-	>.250	.36	.13 – 1.00	.049*	.32	.07 – 1.53	.153
Methotrexate	-	-	>.250	.13	.02 – 1.02	.053	.33	.10 – 1.11	.074
Hydroxychloroquine	-	-	>.250	.45	.18 – 1.13	.088	.25	.05 – 1.21	.085
Age of debut (pr year)	1.03	1.00 – 1.05	.075	1.03	1.00 – 1.06	.045*	-	-	>.250
Negative anti-RNP antibodies	-	-	>.250	-	-	>.250	2.96	.78 – 11.27	.111
Anti-U1-70 kDa positive	-	-	>.250	-	-	>.250	.31	.09 – 1.10	.069
Anti-U1-A positive	-	-	>.250	-	-	>.250	.34	.09 – 1.20	.094

[1]includes patients in remission both on and off therapy – medications allowed: hydroxychloroquine, proton pump inhibitors, calcium channel blockers, intermittent use of NSAID's, low-dose oral corticosteroids (⩽ 5 mg daily) and stable maintenance doses of azathioprine, methotrexate and mycophenolate. [2] Ever present at T1, * significant, **highly significant, CK: Creatine Kinase, FVC: Forced Vital Capacity, NSAID's: Non steroidal anti -inflammatory drugs, anti-RNP: anti-ribonucleoprotein, OR: Odds Ratio, CI: Confidence Interval, T1: Time point 1
